# P-782. Developing a Multivariable Prediction Model of Antibiotic Heteroresistance to First-Line Therapies for Urinary Tract Infection

**DOI:** 10.1093/ofid/ofaf695.993

**Published:** 2026-01-11

**Authors:** Sarah K Blaine, Sarah Lohsen, Julia A Van Riel, Madeleine Boulis, Alexandra C Rios, D’Ante Gooden, Gillian Smith, Paulina Rebolledo, Lucy S Witt, David Weiss, Sarah W Satola, Jessica Howard-Anderson

**Affiliations:** Emory University School of Medicine, Atlanta, GA; Emory University School of Medicine, Atlanta, GA; Northwestern University Feinberg School of Medicine, Chicago, Illinois; Emory University School of Medicine, Atlanta, GA; Emory University, Pike Road, Alabama; Emory University School of Medicine, Atlanta, GA; Georgia Emerging Infections Program, Decatur, Georgia; Emory University School of Medicine, Emory University Rollins School of Public Health, Atlanta, GA; Emory University, Pike Road, Alabama; Emory University, Pike Road, Alabama; Emory University School of Medicine, Division of Infectious Diseases, Atlanta, Georgia; Emory University, Pike Road, Alabama

## Abstract

**Background:**

Heteroresistance (HR), where a subpopulation of bacteria is phenotypically resistant while most of the population appears susceptible, is not routinely tested for despite its potential association with antibiotic failure. Understanding clinical predictors of HR may improve antibiotic selection. Thus, we aimed to develop and validate a model predicting HR to oral antibiotics for urinary tract infection (UTI) in urinary *Escherichia coli* isolates.

**Figure:**
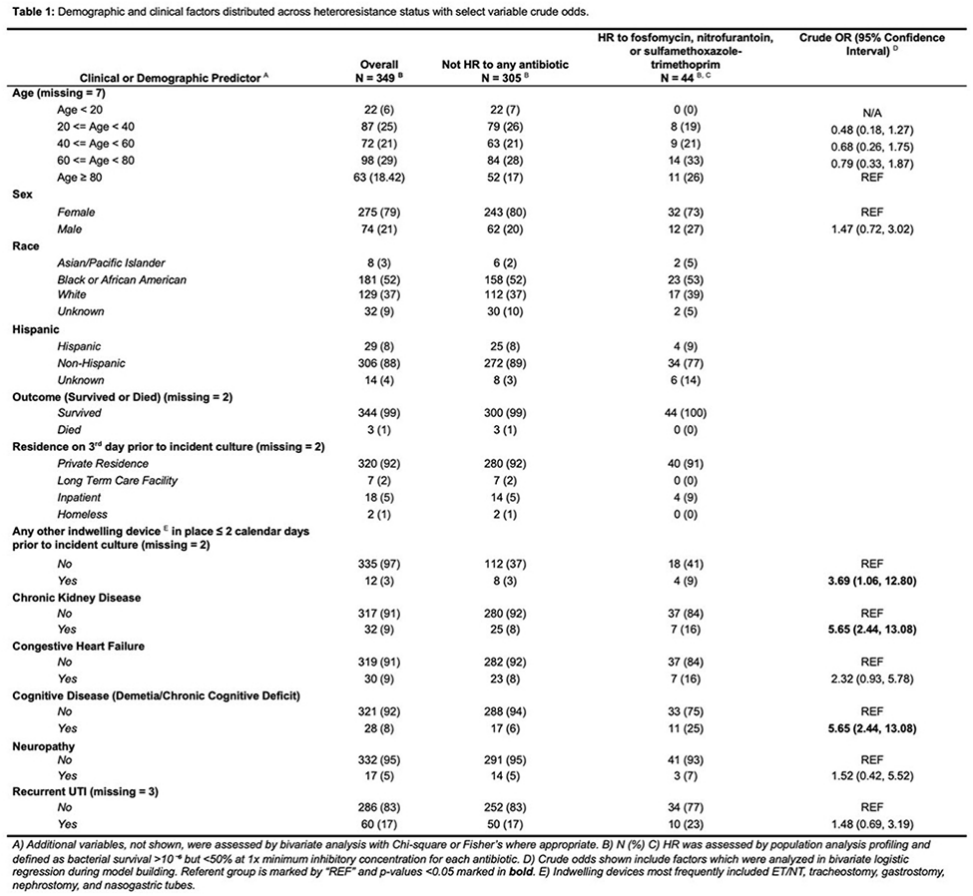

**Figure:**
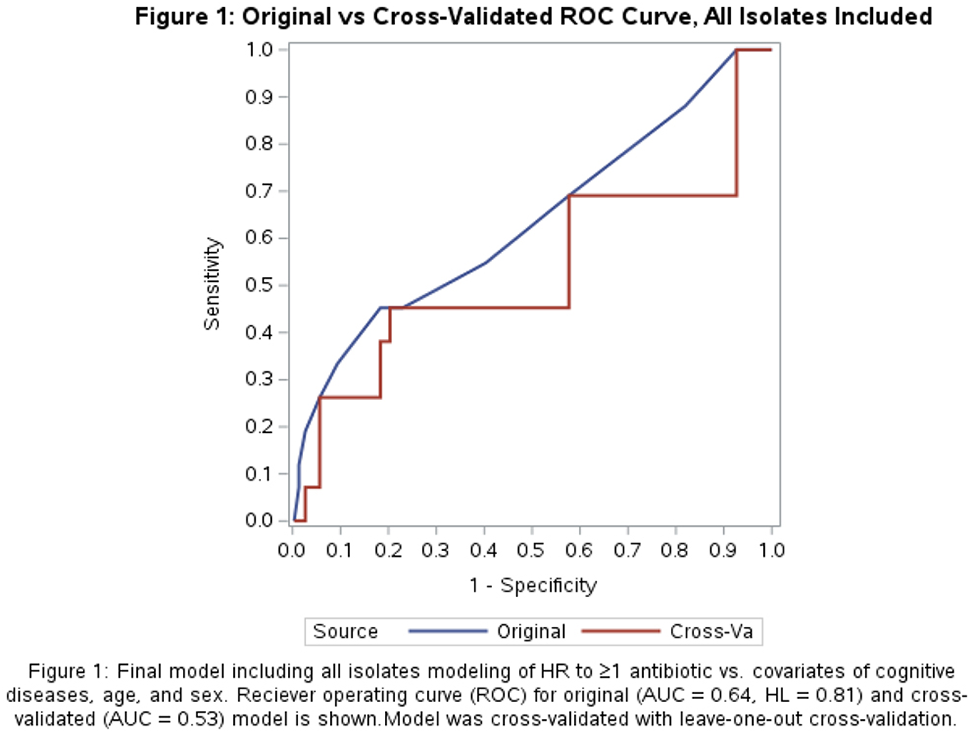

**Methods:**

We analyzed urinary *E. coli* isolates collected during a surveillance pilot performed by the CDC-funded Georgia Emerging Infections Program. HR to nitrofurantoin, fosfomycin, or sulfamethoxazole-trimethoprim was assessed by population analysis profiling (PAP) and defined as bacterial survival >10^-6^ but < 50% at 1x minimum inhibitory concentration. Covariates were collected by chart review. We used logistic regression with forward selection to build models predicting HR to ≥1 antibiotic, assessed performance using area under the curve (AUC) and Hosmer-Lemeshow (HL) goodness-of-fit, and performed n-fold cross-validation in SAS. All models controlled for age and sex. A subgroup analysis excluded isolates resistant by PAP (comparing susceptible to HR).

**Figure:**
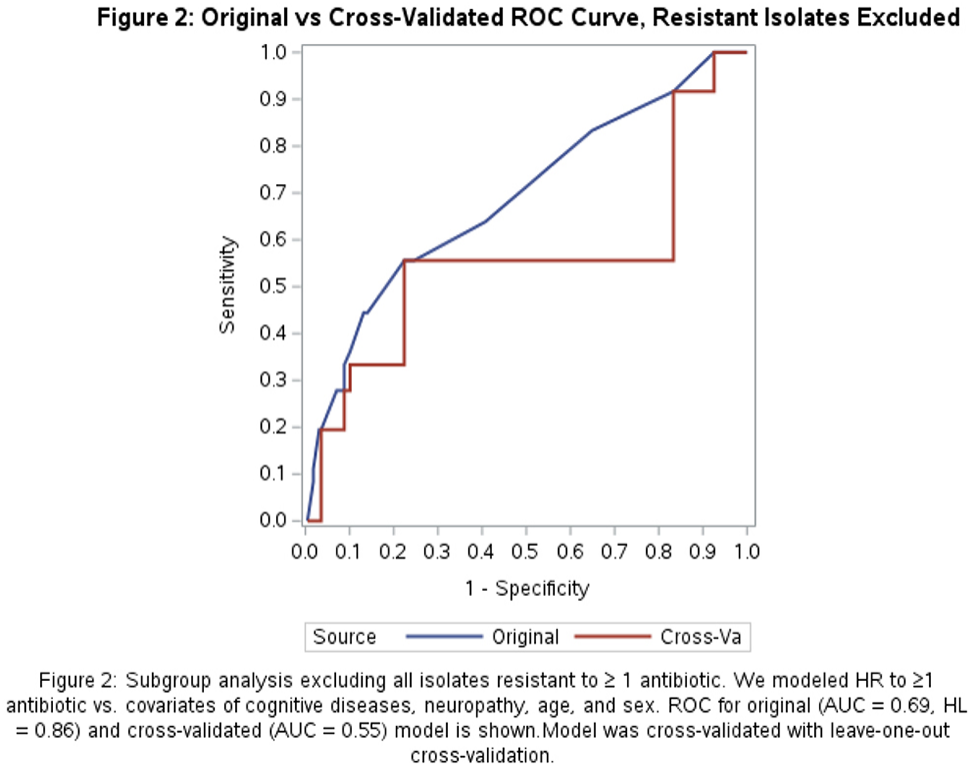

**Results:**

Of 349 isolates included, 275 (79%) were from females with median age 56 (Table 1). Forty-four (13%) were HR to ≥1 antibiotic. In unadjusted analyses, an indwelling device in place prior to collection, chronic kidney disease, and cognitive disease were significantly associated with HR to ≥ 1 antibiotic (Table 1). The final multivariable model included cognitive disease, age, and sex (AUC = 0.64, HL = 0.81) (Figure 1). After cross-validation the mean AUC was 0.53. In a subgroup analysis excluding resistant isolates, 38/271 (14%) were HR to ≥1 antibiotic. In this subgroup, the model predicting HR to ≥1 antibiotic included cognitive disease, neuropathy, age, and sex (AUC = 0.69, HL = 0.86). Cross-validation yielded a mean AUC of 0.55 (Figure 2).

**Conclusion:**

We created a novel model using clinical risk factors to predict HR to first-line antibiotics used for UTIs. This model provided modest prediction of HR but may have limited generalizability when used on other populations. Future work should increase sample size for model development and validate on outside populations.

**Disclosures:**

Lucy S. Witt, MD, MPH, Merck & Co: Grant/Research Support

